# Blood phenylalanine instability strongly correlates with anxiety in phenylketonuria

**DOI:** 10.1016/j.ymgmr.2017.12.003

**Published:** 2017-12-27

**Authors:** Bozena Didycz, Miroslaw Bik-Multanowski

**Affiliations:** Department of Medical Genetics, Jagiellonian University Medical College, Krakow, Poland

**Keywords:** Adolescents, Neuropsychiatric symptoms, PKU, Quality of life, Treatment adherence, PKU, phenylketonuria, Phe, phenylalanine, STAI, State Trait Anxiety Inventory

## Abstract

We assessed the relationship between anxiety and long-term metabolic control in adolescents with phenylketonuria (PKU). We used a standardized psychological test to measure anxiety level and analyzed lifelong blood phenylalanine stability in a selected group of 25 PKU teenagers with treatment adherence problems. We demonstrated significant correlations of anxiety with variability of blood phenylalanine concentrations and with severity of hyperphenylalaninemia. Avoiding blood phenylalanine fluctuations in childhood can probably reduce anxiety in PKU adolescents.

## 1 Introduction

Phenylketonuria (PKU; OMIM 261600) is one of the most common inborn errors of metabolism and has become a classic example of a treatable rare disease. A therapeutic diet with low content of phenylalanine (Phe) should be implemented as soon as possible, ideally within the first two weeks of life, to avoid serious brain damage and intellectual disability in affected children [1]. The patients must adhere to the dietary regimen for their entire lives.

Unfortunately, as in other chronic diseases, treatment adherence in PKU decreases with time. This results in frequent occurrence of episodes of high blood Phe concentrations and it may lead to brain dysfunction presenting with neuropsychological deficits and behavioral disturbances [2], [3], [4], [5]. Although serious neuropsychological deficits were frequently reported, especially in adolescents who abandoned dietary treatment, little is known about the prevalence of the less tangible, psychiatric complications such as anxiety and depression, which may jeopardize the therapeutic goals. Previous reports have often been based on clinical observations in adult patients [6], [7], whereas the situation in adolescents is less clear.

In this study we focused on the measurement of anxiety levels in teenagers with PKU using standardized psychological methods. Based on published observations [8], [9] and on our clinical experience with patients from this age group, we hypothesized that anxiety can be related to instability of blood Phe concentrations.

## 2 Material and methods

We invited to our study all PKU teenagers aged 13–17 years, in whom treatment was introduced no later than in the sixth week of life, and who were subsequently continuously followed-up in our clinic. The study entry criteria included also normal intellectual development, treatment adherence problems (at least 25% of the results of control blood Phe tests exceeding the recommended limit of 360 μmol/L), and availability of genotyping results of the phenylalanine hydroxylase gene (both disease-causing mutations identified). A group of 25 PKU patients (18 girls and 7 boys) fulfilled the inclusion criteria and participated in the study.

During a routine follow-up visit, every patient was assessed with use of The State-Trait Anxiety Inventory (STAI), which is an introspective psychological inventory consisting of 40 self-report items pertaining to anxiety affect. The STAI distinguishes between a person's state and trait anxiety levels. State anxiety (A-State) can be defined as fear, nervousness and discomfort that are temporarily induced by situations perceived as dangerous. Trait anxiety (A-Trait) can be defined as a relatively enduring disposition to feel stress, worry and discomfort. Higher STAI scores suggest higher anxiety [10].

Following psychological assessment, blood Phe concentration was measured in every study participant.

In order to analyze the relationship of the STAI scores with dynamics of blood hyperphenylalaninemia we assessed a total of 4039 historical results of blood Phe tests, which were available in our clinic. We considered two indicators of the severity of historical hyperphenylalaninemia: the lifetime mean blood Phe concentration and the recent levels of hyperphenylalaninemia prior to psychological testing. In addition, we analyzed lifetime standard deviation of blood Phe results, reflecting the metabolic stability in a given patient, and the frequency of blood Phe measurements – an indicator of treatment adherence.

The Pearson correlation statistics and multiple regression analysis were used for data analysis. Benjamini-Hochberg FDR correction for multiple comparisons was applied to confirm the statistical significance of the observed correlations.

All procedures were in accordance with the 1964 Helsinki Declaration and Good Clinical Practice guidelines. The regional ethics committee approved the study. Informed consent was obtained from all study participants.

## 3 Results

Table 1 presents demographic details, metabolic and psychological data of the study participants.

**Table 1:** The participants of the study, their metabolic profiles and assessment of anxiety with use of The State-Trait Anxiety Inventory (STAI).

Patient	Age (years)	Gender	Genotype (*PAH* gene mutations)	IQ	Treatment start (week of life)	Mean, lifetime blood Phe concentration (mmol/L)	Blood Phe concentration on the day of psychological assessment (mmol/L)	SD of Phe concentration (mmol/L)	Frequency of blood testing (mean yearly number of tests)	STAI scores	
										A-Trait (percentile)	A-State (percentile)
1	14	F	p.[Arg408Trp];[Arg408Trp]	93	5	0.56	1.13	0.38	12	41	53
2	14	F	p.[Arg408Trp];[Arg408Trp]	110	4	0.49	0.49	0.39	11	50	20
3	14	F	p.[Arg408Trp];[Arg408Trp]	123	3	0.4	0.60	0.49	9	35	20
4	14	M	p.[Arg408Trp];[Arg243Gln]	108	3	0.28	0.25	0.25	18	16	19
5	13	F	p.[Arg408Trp];[Arg408Trp]	127	3	0.29	0.27	0.36	9	23	89
6	14	F	p.[Arg408Trp];[Arg408Trp]	97	4	0.72	1.37	0.52	9	64	53
7	13	F	p.[Arg408Trp];[Arg408Trp]	89	6	0.35	0.78	0.25	11	6	26
8	14	F	p.[Arg408Trp];[Glu221_Asp222 > Glufs]	116	3	0.62	0.51	0.32	13	18	14
9	15	F	p.[Arg408Trp];c.[1066-11G > A]	109	3	0.7	0.39	0.40	6	37	23
10	14	M	p.[Arg408Trp];[Arg408Trp]	112	3	0.67	1.07	0.38	15	30	52
11	13	F	p.[Arg408Trp];[Arg408Trp]	106	2	0.77	1.31	0.38	12	59	68
12	14	F	p.[Arg408Trp];[Arg408Trp]	90	4	0.63	0.82	0.35	10	13	49
13	14	F	p.[Arg408Trp];[Ile283Phe]	87	2	0.67	0.91	0.40	7	68	63
14	15	F	p.[Arg408Trp];[Arg408Trp]	102	3	0.65	1.46	0.33	8	79	57
15	13	M	p.[Arg408Trp];c.[1315 + 1G > A]	105	3	0.37	0.28	0.35	18	19	62
16	15	F	p.[Arg408Trp];[Arg408Trp]	85	4	0.52	0.45	0.25	15	5	23
17	15	M	p.[Arg408Trp];[Arg408Trp]	101	5	0.44	0.37	0.32	13	5	1
18	13	F	p.[ArgR408Trp];[Tyr414Cys]	109	3	0.37	0.12	0.31	10	13	79
19	15	F	p.[Arg408Trp];[Arg408Trp]	108	4	0.41	0.98	0.41	8	23	1
20	17	M	p.[Arg408Trp];[Arg408Trp]	96	4	0.65	1.06	0.38	6	25	15
21	17	M	p.[Arg408Trp];[Arg408Trp]	117	3	0.48	0.6	0.25	6	12	6
22	17	F	p.[Arg408Trp];[Arg408Trp]	94	6	0.61	0.88	0.34	6	28	25
23	14	M	p.[Arg408Trp];[Arg408Trp]	124	3	0.37	0.53	0.29	18	25	41
24	16	F	p.[Arg408Trp];[Arg408Trp]	87	6	0.39	0.82	0.26	11	15	10
25	16	F	p.[Arg408Trp];[Gly188 > Alafs]	132	3	0.32	0.4	0.28	14	23	56

Mean (min–max)						0.51 (0.28–0.77)	0.69 (0.12–1.46)	0.35 (0.25–0.52)	11 (6–18)	29 (5–79)	37 (1–89)

The analysis of relationship of the STAI results with the severity of hyperphenylalaninemia revealed moderate-to-strong correlations of lifetime mean blood Phe concentrations and of recent blood Phe levels with A-Trait scores (Pearson correlation coefficient *r* = 0.59 and 0.69, corrected *p* = 0.005 and 0.0012, respectively). The assessment of relationship between long-term stability of blood Phe, represented by the SD values, and anxiety levels also resulted in detection of a statistically significant correlation (*r* = 0.60; corrected *p* = 0.005). No statistically significant findings were detected with regard to the mean frequency of blood Phe measurements in relation with A-Trait scores (*r* = 0.31) as well as for the A-State scores in relation to all analyzed metabolic parameters.

Next, multiple regression analysis was performed to assess the combined influence of recent blood Phe concentrations and of blood Phe standard deviation values on the anxiety level. The model was statistically significant (multiple R^2^ = 0.438, *p* = 0.0017) and revealed stronger correlation of SD of blood Phe values with A-Trait scores (standardized regression weight B = 0.503, *p* = 0.007) in comparison with recent blood Phe concentrations (B = 0.294, *p* = 0.09).

## 4 Discussion

Only few interventional studies have assessed neuropsychiatric symptoms that affect quality of life in PKU [6]. The assessed populations differed in regard to important aspects, such as treatment initiation time, age, standards of PKU treatment (different recommendations in various countries), and length of observation. We assessed a relatively small but clinically homogenous group of continuously treated teenagers, and we analyzed their entire treatment history.

The feeling of anxiety may develop in suddenly occurring stressful situations or may be linked to longer-lasting psychological disorders. The STAI allows for differentiation between both conditions. Our results suggest that A-Trait scores reflect the brain effects of hyperphenylalaninemia better than A-State scores. This can probably be explained by relatively low dynamics of hyperphenylalaninemia in PKU patients, who usually require several weeks to normalize high blood Phe concentration. An increase of A-State score refers more to how a person is feeling at the time of a perceived threat and is considered temporary [10]. Based on our results it can be hypothesized that the follow-up visit itself is not perceived by PKU teenagers as a big threat even if they expect that their blood Phe levels exceed the recommended limits.

Short-term increase in Phe concentrations limits the transport of tyrosine and tryptophan across the blood-brain barrier [11], which in turn directly affects the brain production of dopamine and serotonin [12], [13]. This can result in, at least partially reversible, neuropsychological abnormalities and decreased mood [3], [4], [5]. However, prolonged hyperphenylalaninemia can additionally decrease cerebral protein synthesis [14] and result in dysmyelination [15] with, potentially permanent, serious brain damage [16], [17]. Our findings suggest that disposition to feel stress and worry in PKU teenagers can be a long-lasting and hardly reversible phenomenon. It seems also that increased anxiety level can result from hyperphenylalaninemia, which is in agreement with previous observations [9], and from high changeability of blood Phe concentration.

In conclusion, every effort should be made by physicians, psychologists and dieticians taking care of patients with PKU to avoid not only hyperphenylalaninemia but also large fluctuations of blood Phe in affected persons since early childhood. Continuous support to PKU families and high-quality metabolic control in children can result in lower frequency of anxiety, better quality of life and, possibly, better cooperation with affected adolescents, who are going through their teenage rebellion period.

## Conflicts of interest

The authors declare no conflicts of interests.

## Funding

This work was supported by the Nutricia Research Foundation (grant number 02/2011).

## References

[1] van Spronsen F.J., van Wegberg A.M., Ahring K. (2017). Key European guidelines for the diagnosis and management of patients with phenylketonuria. Lancet Diabetes Endocrinol..

[2] Walter J.H., White F.J., Hall S.K. (2002). How practical are recommendations for dietary control in phenylketonuria?. Lancet.

[3] Bik-Multanowski M., Didycz B., Mozrzymas R. (2008). Quality of life in noncompliant adults with phenylketonuria after resumption of the diet. J. Inherit. Metab. Dis..

[4] Bik-Multanowski M., Pietrzyk J.J., Mozrzymas R. (2011). Routine use of CANTAB system for detection of neuropsychological deficits in patients with PKU. Mol. Genet. Metab..

[5] Didycz B., Nitecka M., Bik-Multanowski M. (2017). The use of d2 and benton tests for assessment of attention deficits and visual memory in teenagers with phenylketonuria. J. Inherit. Metab. Dis. Rep..

[6] Bilder D.A., Noel J.K., Baker E.R. (2016). Systematic review and meta-analysis of neuropsychiatric symptoms and executive functioning in adults with phenylketonuria. Dev. Neuropsychol..

[7] Pietz J., Fätkenheuer B., Burgard P. (1997). Psychiatric disorders in adult patients with early-treated phenylketonuria. Pediatrics.

[8] Stemerdink B.A., Kalverboer A.F., van der Meere J.J. (2000). Behaviour and school achievement in patients with early and continuously treated phenylketonuria. J. Inherit. Metab. Dis..

[9] Manti F., Nardecchia F., Chiarotti F. (2016). Psychiatric disorders in adolescent and young adult patients with phenylketonuria. Mol. Genet. Metab..

[10] Spielberger C.D., Sydeman S.J., Maruish M.E. (1994). State-trait anxiety inventory and state-trait anger expression inventory. The Use of Psychological Testing for Treatment Planning and Outcome Assessment.

[11] Choi T.B., Pardridge W.M. (1986). Phenylalanine transport at the human blood-brain barrier. Studies with isolated human brain capillaries. J. Biol. Chem..

[12] Burlina A.B., Bonafé L., Ferrari V. (2000). Measurement of neurotransmitter metabolites in the cerebrospinal fluid of phenylketonuric patients under dietary treatment. J. Inherit. Metab. Dis..

[13] Landvogt C., Mengel E., Bartenstein P. (2008). Reduced cerebral fluoro-L-dopamine uptake in adult patients suffering from phenylketonuria. J. Cereb. Blood Flow Metab..

[14] Hoeksma M., Reijngoud D.J., Pruim J. (2009). Phenylketonuria: high plasma phenylalanine decreases cerebral protein synthesis. Mol. Genet. Metab..

[15] Cleary M.A., Walter J.H., Wraith J.E. (1995). Magnetic resonance imaging in phenylketonuria: reversal of cerebral white matter change. J. Pediatr..

[16] Enns G.M., Koch R., Brumm V. (2010). Suboptimal outcomes in patients with PKU treated early with diet alone: revisiting the evidence. Mol. Genet. Metab..

[17] Daelman L., Sedel F., Tourbah A. (2014). Progressive neuropsychiatric manifestations of phenylketonuria in adulthood. Rev. Neurol..

